# Unlocking the future of deep brain stimulation: innovations, challenges, and promising horizons

**DOI:** 10.1097/JS9.0000000000001279

**Published:** 2024-03-04

**Authors:** Nour Shaheen, Oliver Flouty

**Affiliations:** a Alexandria University, Alexandria Faculty of Medicine, Alexandria, Egypt; b Department of Neurosurgery and Brain Repair, University of South Florida, Tampa, Florida

Deep brain stimulation (DBS) is a surgical intervention wherein electrodes are surgically implanted into precise brain regions to regulate aberrant neural activity. This approach is employed for the treatment of a range of neurological and psychiatric conditions that are unresponsive to conventional therapeutic methods. Globally, it is estimated that more than 160 000 patients have undergone DBS for diverse neurological conditions^1^. However, it is noteworthy that the utilization of DBS for psychiatric indications worldwide is relatively limited, likely involving fewer than 500 cases^2^. Notably, in the United States, there has been a consistent upward trend in the annual number of DBS procedures, with admissions exceeding 5500 patients^3^.

DBS has gained FDA approval for various indications over the years. In 1997, FDA approval was granted for essential tremor, targeting the ventral intermediate nucleus (VIM) of the thalamus. This was followed by approval for Parkinson’s disease in 2002, with the subthalamic nucleus (STN) or the globus pallidus internus (GPi) as target areas. In 2003, GPi DBS FDA approval for dystonia was granted under humanitarian device exemption (HDE). Subsequently, Obsessive-Compulsive Disorder (OCD) received FDA approval for DBS in 2009 as a humanitarian use device (also under HDE), targeting the ventral capsule/ventral striatum (VC/VS)^4^. Finally, in 2018, DBS gained FDA approval for Epilepsy, with the anterior nucleus of the thalamus designated as the target area^5^ (Fig. 1).

**Figure 1:**
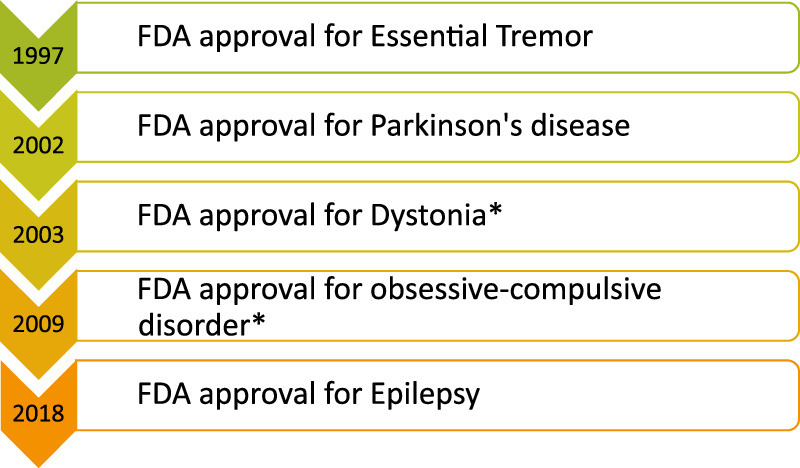
FDA approval for various neurological and psychiatric disorders following a chronological order. *Food and Drug Administration (FDA) approval under the category of Humanitarian Device Exemption (HDE).

Nonetheless, the scope of DBS applications is continually expanding, with ongoing clinical trials and research exploring its potential effectiveness for conditions such as Alzheimer’s disease, depression, Tourette syndrome, and chronic pain.

The effectiveness and safety of DBS therapy, in comparison to alternative medical treatments or neurological procedures, can exhibit variability contingent on factors such as the specific disorder, the targeted brain region, the chosen stimulation parameters, and the unique characteristics of each patient^6^. Notably, recent advancements in DBS technology have been directed at enhancing the therapeutic efficacy and safety of DBS. One such innovation is adaptive DBS (aDBS), a technique incorporating a feedback mechanism to dynamically adjust stimulation parameters based on recorded brain signals from implanted electrodes^6^. This personalized and precise modulation of impaired motor networks has shown promise in Parkinson’s Disease, reducing stimulation duration, energy consumption, and ameliorating motor outcomes while mitigating DBS-related side effects. Nevertheless, it is imperative to acknowledge that aDBS remains an experimental approach necessitating further validation and optimization. Challenges and limitations associated with aDBS implementation encompass the selection of optimal brain signals and feedback control algorithms, ensuring the device’s reliability and safety, and assessing the long-term effects and advantages of aDBS. Currently, several ongoing clinical trials are aimed at evaluating the safety and initial efficacy of long-term aDBS in PD patients through the utilization of a new generation of DBS devices^6^.

However, DBS is not without risks and limitations. Some of the potential complications of DBS include infection, bleeding, hardware malfunction, and stimulation-induced adverse effects. DBS may also cause cognitive, emotional, and behavioral changes in some patients, depending on the target location and stimulation parameters. Therefore, careful patient selection, preoperative evaluation, and postoperative management are essential for optimizing the safety and efficacy of DBS.

Ongoing research and clinical trials are exploring the use of DBS for various medical conditions. In the context of Parkinson’s disease, DBS is being investigated to alleviate movement-related symptoms such as tremors, stiffness, and slowness. Trials include the Phase I DBS vs. Best Medical Therapy (BMT) Trial, which compares DBS to medical therapy and different brain target areas, and a trial evaluating an adaptive DBS system for improved symptom management^7,8^.

For depression, DBS is being studied as a means to enhance mood and quality of life by targeting specific brain areas involved in emotion regulation. Trials comprise the DBS Therapy for Treatment Resistant Depression, focusing on the medial forebrain bundle, and the Efficacy and Safety of Dual-target DBS for Treatment-resistant Alcohol Use Disorder, targeting the nucleus accumbens and anterior limb of the internal capsule^9,10^.

In cases of stroke, where blood flow interruption results in brain damage and functional impairment, DBS is explored for recovery and rehabilitation by stimulating brain regions involved in motor control and learning. Trials include the assessment of cerebellar poststroke DBS for upper limb function improvement and DBS applied to the dentate nucleus to enhance arm function in stroke patients. These studies contribute to expanding the potential applications and effectiveness of DBS for a range of medical conditions^11^.

The future of DBS in neurosurgery holds great promise, driven by advancing technology and expanding research into novel applications and outcomes. Key avenues for enhancing the potential of DBS include:

- *Technological advancements* such as miniaturization of implants, resulting in smaller, more flexible electrodes and devices that reduce invasiveness, lower the risk of infection, and enhance patient comfort and aesthetics. Additionally, longer-lasting batteries, rechargeable and wireless, extend device lifespans, reduce the need for replacements, and boost patient convenience and compliance.
- *Advanced device capabilities*, including programmability for precise and individualized stimulation parameters, hold the potential to improve DBS efficacy, safety, and adaptability to changing clinical conditions. The use of robotics in surgical planning and execution enhances accuracy, speed, and electrode placement reliability while minimizing human error and radiation exposure. In addition to these technological strides, DBS explores new target areas and indications across various functions and disorders, such as stroke recovery, treatment-resistant depression, and conditions like chronic pain, addiction, obesity, Alzheimer’s disease, traumatic brain injury, and autism.
- *Innovative techniques* involve less invasive approaches with smaller, flexible electrodes and devices that improve patient comfort and safety, along with enhanced efficacy and safety through precise, programmable settings and adaptive DBS, which can adjust to a patient’s clinical condition or feedback. Directional leads (D-leads) enable precise targeting of specific brain regions while avoiding unwanted areas, while sensing electrodes record brain activity and adjust stimulation parameters to create a closed-loop system. Variable-frequency stimulation modulates abnormal neural activity and restores normal brain network function, and remote programming and telemedicine platforms allow patients to adjust settings from home and receive online support from healthcare providers. Robotic stereotactic assistance further enhances electrode placement accuracy and reduces the need for awake surgery, while combination therapies combining DBS with gene therapy, stem cell therapy, or pharmacotherapy offer the potential to enhance benefits and slow the progression of neurodegenerative diseases.

DBS raises ethical concerns as it advances:

Personal Identity and Autonomy: DBS may impact patients’ identity and decision-making^12^. Ensuring respect for dignity and integrity while avoiding compromise of authenticity is crucial. aDBS Risks: The benefits of aDBS, with automatic stimulation adjustments, include precision and personalization^12^. However, concerns about data privacy and patient control over settings must be addressed. Cognitive Enhancement Dilemmas: The potential for DBS to enhance cognitive abilities prompts ethical questions about fairness, access, and regulation. Preventing misuse and addressing social inequalities are paramount^12^.

These ethical considerations, though not exhaustive, emphasize the need for ongoing reflection and dialogue among stakeholders in the evolving field of DBS.

## Ethical approval

Ethics approval was not required for this review.

## Consent

Informed consent was not required for this review.

## Sources of funding

None.

## Author contribution

N.S. and O.F: conceptualization, methodology, writing – original draft preparation, and writing –reviewing and editing. All authors approved the final manuscript.

## Conflicts of interest disclosure

All authors declares no conflicts of interest.

## Research registration unique identifying number (UIN)

Not applicable.

## Guarantor

Not applicable.

## Data availability statement

The journal requires authors to include in any articles that report results derived from research data to include a data availability statement. Please confirm if any datasets generated during and/or analyzed during the current study are publicly available, available upon reasonable request, or if data sharing is not applicable to this article.

## Provenance and peer review

Not applicable.
